# Solubility of lamotrigine in age-specific biorelevant media that simulated the fasted- and fed-conditions of the gastric and intestinal environments in pediatrics and adults: implications for traditional, re-formulated, modified, and new oral formulations

**DOI:** 10.1186/s12896-023-00809-2

**Published:** 2023-09-08

**Authors:** Ramzi Shawahna, Hala Saba’aneh, Amal Daraghmeh, Yara Qassarwi, Valentina Franco, Xavier Declèves

**Affiliations:** 1 Department of Physiology, Pharmacology and Toxicology, Faculty of Medicine and Health Sciences, An-Najah National University, New Campus, Building: 19, Office: 1340, P.O. Box 7, Nablus, Palestine; 2 Clinical Research Center, An-Najah National University Hospital, Nablus, 44839 Palestine; 3 Department of Pharmacy, Faculty of Medicine and Health Sciences, An-Najah National University, Nablus, Palestine; 4 Section of Translational Neurovascular Research, IRCCS Mondino Foundation, Via Mondino 2, Pavia, 27100 Italy; 5 Clinical and Experimental Pharmacology Unit, Department of Internal Medicine and Therapeutics, University of Pavia, Pavia, 27100 Italy; 6 Biologie du Médicament-Toxicologie, AP-HP, Hôpital Cochin, 27 rue du Faubourg St. Jacques, Paris, 75679 France; 7 Faculty of Health, Université Paris Cité, Inserm, UMRS-1144, Optimisation Thérapeutique en Neuropsychopharmacologie, Paris, 75006 France

**Keywords:** Lamotrigine, Pediatric, Solubility, Dosage form performance, Biopharmaceutics, Biorelevant

## Abstract

**Background:**

Lamotrigine is an effective antiseizure medication that can be used in the management of focal and generalized epilepsies in pediatric patients. This study was conducted to quantify and compare the solubility of lamotrigine in age-specific biorelevant media that simulated the fasted and fed conditions of the gastric and intestinal environments in pediatrics and adults. Another aim was to predict how traditional, re-formulated, modified, and new oral formulations would behave in the gastric and intestinal environments across different age groups.

**Methods:**

Solubility studies of lamotrigine were conducted in 16 different age-specific biorelevant media over the pH range and temperature specified by the current biopharmaceutical classification system-based criteria. The age-specific biorelevant media simulated the environments in the stomach and proximal gastrointestinal tract in both fasted and fed conditions of adults and pediatric sub-populations. The solubility of lamotrigine was determined using a pre-validated HPLC-UV method.

**Results:**

Lamotrigine showed low solubility in the 16 age-specific biorelevant media as indicated by a dose number of > 1. There were significant age-specific variabilities in the solubility of lamotrigine in the different age-specific biorelevant media. Pediatric/adult solubility ratios of lamotrigine fell outside the 80-125% range in 6 (50.0%) and were borderline in 3 (25.0%) out of the 12 compared media. These ratios indicated that the solubility of lamotrigine showed considerable differences in 9 out of the 12 (75.0%) of the compared media.

**Conclusion:**

Future studies are still needed to generate more pediatric biopharmaceutical data to help understand the performances of oral dosage forms in pediatric sub-populations.

**Supplementary Information:**

The online version contains supplementary material available at 10.1186/s12896-023-00809-2.

## Introduction

The biopharmaceutical classification system (BCS) has been used to support and hasten the development of traditional, new, and/or modified immediate-release solid oral dosage forms (IRSODFs) [1]. Drug regulatory agencies like the European Medicines Agency (EMA), the US Food and Drug Administration (FDA), and the World Health Organization (WHO) use the BCS to allow waiving in vivo bioequivalence studies (IVBESs) (biowaiver) for IRSODFs that contain highly soluble active pharmaceutical ingredients (APIs) [2–6]. These biowaiver decisions allow pharmaceutical scientists to surrogate IVBESs by in vitro dissolution tests. Therefore, biowaiver decisions can reduce the costs of developing IRSODFs, accelerate regulatory processing and approval, and reduce the regulatory burden [7]. It is important to note that the principles guiding IVBESs in pediatrics were not developed [6, 8–11]. Additionally, there are recognizable variabilities in the quantities and qualities of the gastrointestinal fluids between adult and pediatric populations [6, 11, 12]. Moreover, the volume and composition of the gastrointestinal fluids vary by age, development, and maturation. Therefore, pediatrics can be divided into subpopulations that include: neonates, infants, children, and adolescents [6, 11, 12].

The permeability and solubility of APIs can vary by age, developmental status, and maturation. Recently, there has been a growing interest in studying how the permeability and solubility of APIs can change using age-specific criteria for the volume and constituents of the gastrointestinal fluids [8–11, 13–18]. Previous studies have classified the drugs listed by the WHO as essential drugs for adults and children into provisional BCS classes [6, 9, 19–23]. A study conducted by Charoo et al. assessed the possible harms associated with expanding the BCS-based biowaiver standards for fluconazole to subgroups of children [24]. In another study, variabilities in the solubility of celecoxib in different age-specific pediatric and adult biorelevant media were assessed [11].

Since its approval in different countries, lamotrigine has become one of the most commonly used antiseizure medications. Lamotrigine is one of the first-line treatments for Lennox-Gestault syndrome, focal-onset tonic-clonic seizures, and simple and complex partial seizures [25, 26]. In clinical practice, lamotrigine is also used for prophylaxis of basilar migraine, panic disorder, rapid-cycling bipolar depression, and binge eating disorder [27]. Additionally, lamotrigine has been suggested to be useful in the management of trigeminal neuralgia [28].

Traditional, re-formulated, and novel IRSODFs containing lamotrigine as the API are needed for adult and pediatric patients. A recent study explored the possibility of extrapolating adult lamotrigine solubility from adults to children [29]. However, the study used a limited number of biorelevant media and did not account for the different types of feed used in pediatric populations including breast milk, cow milk-based formulas, soy-based formulas, and a phenylalanine-free formula. Therefore, little studies were conducted to assess variabilities in the solubility of lamotrigine in different age-specific biorelevant media. Similarly, the potential effects of these solubility differences on the performance of IRSODFs containing lamotrigine were not assessed before [30, 31]. Therefore, this study was done to quantify and compare the solubility of lamotrigine in 16 age-specific biorelevant media that simulated the fasted and fed conditions of the gastric and intestinal environments in pediatrics and adults. Another aim was to predict how traditional, re-formulated, modified, and new IRSODFs would behave in the gastric and intestinal environments across different age groups.

## Methods

### Materials

Acetonitrile, egg lecithin, sodium hydroxide, acetic acid, pepsin, maleic acid, glyceryl monooleate, hydrochloric acid, sodium oleate, sodium taurocholate, sodium hydroxide, sodium chloride, sodium acetate, and methanol were obtained from Sigma-Aldrich Chemie GmbH (Taufkirchen, Germany). Lamotrigine was a gift from Jerusalem Pharmaceuticals Co. Ltd. HPLC grade water was used in this study. Dialysis tubes with molecular weight cutoffs of 12,000–14,000 Da were bought from Medicell Membranes Ltd., London, UK. For the preparation of all biorelevant media, ultra-pure (Milli-Q) water was used. Adult fed-condition biorelevant media were prepared using whole cow milk. The cow milk was pre-treated using ultra-high temperature and contained < 4% fat. The pediatric fed-condition media were prepared using three infant formulas: (1) cow milk-based formula (S-26® One Infant Formula, 0–6 months, Wyeth Nutrition), (2) soya-based formula (Similac® Isomil 1, 0–6 months, Abbott), and (3) phenylalanine-free formula for infants and toddlers with phenylketonuria (Phenyl-Free® 1, infant-toddler, Mead Johnson Nutrition). The instruments used included Agilent Technologies 1200 series HPLC system (Santa Clara, CA), pH meter, and borosilicate glass tubes.

### Development of the age-specific biorelevant media

The 16 different age-specific biorelevant media that simulated the environments in the stomach and proximal gastrointestinal tract in fasted and fed conditions of adults and pediatric sub-populations were prepared as previously described [11, 12, 32]. The recipe used to prepare each of the biorelevant media is shown in Supplementary Table S1. Variabilities in osmolality, pH range, pepsin, lecithin, bile salt, buffering capacity, fat digestion products, and type of feed were accounted for. When needed, the pH of the media was adjusted using sodium bicarbonate or hydrochloric acid. On the other hand, the osmolality was adjusted using sodium chloride, as needed.

### Evaluation of solubility of lamotrigine in the age-specific biorelevant media

Solubility studies were performed to assess the solubility of lamotrigine in the 16 different age-specific biorelevant media.

#### Solubility of lamotrigine in aqueous-based media

To each of the 16 age-specific biorelevant media, excessive amounts of lamotrigine that were sufficient to oversaturate 10 mL were added in borosilicate glass tubes [11, 12, 24]. The tubes were then covered with parafilm and placed in a shaking water bath. Tubes were then shaken in a shaking water bath that was set at 200 strokes/min at 37 ± 0.5 °C for a dwell period of 48 h to ensure equilibrium solubility. Before analysis, each sample was centrifuged at 8,000 rpm for 15 min. The temperature of the centrifuge was set at 37 ± 0.5 °C. The supernatants were aspired and filtered through 0.45 μm regenerated cellulose filters. Each filtrate was then mixed with methanol in a 1:1 ratio before analysis.

#### Solubility of lamotrigine in media containing milk or formula

The fed-conditions media contained milk or formula. Because these media existed as complex multiphase systems, the presence of proteins would impede the filtration of these samples through the 0.45 μm filters [12, 33]. Therefore, the solubility of lamotrigine in the fed-conditions media was evaluated by using equilibrium dialysis to negate the need for direct filtration. Aliquots (5 mL) of freshly prepared media were placed in dialysis membranes (molecular weight cutoff of 12,000 to 14,000 Da). The dialysis membranes containing 5 mL of freshly prepared media were placed in plastic centrifuge tubes. To each plastic centrifuge tube, 20 mL of the respective milk or formula-containing media was added. This volume ensured the submersion of the dialysis membranes containing 5 mL of freshly prepared media [11, 12, 24]. Excessive amounts of lamotrigine that were sufficient to oversaturate 25 mL of each milk or formula-containing media were added to each plastic centrifuge tube. The tubes were then closed and placed in a shaking water bath that was set at 200 strokes/min at 37 ± 0.5 °C. A pilot study was conducted to evaluate the equilibrium solubility of lamotrigine following 72 and 96 h dwell periods. The equilibrium solubility was not different following 72 or 96 h dwell periods. Therefore, the equilibrium solubility of lamotrigine in all fed-conditions biorelevant media was evaluated at a dwell period of 72 h. The dialysis membranes were removed and washed with fresh media. From each dialysis membrane, the contents were taken into Eppendorf tubes and diluted in methanol in a 1:1 ratio. The mixture was vortexed for 10 s.

#### Gastric volume in adults

In IVBESs, IRSODFs are swallowed by adult volunteers with the help of a glass of 250 mL of water [2, 3, 34]. Therefore, APIs are considered highly soluble by the EMA, FDA, and WHO when their maximal dose strength can be dissolved in 250 mL of aqueous-based media in the pH range of 1.2–6.8 at 37 ºC [2, 3, 34–36]. Therefore, the initial gastric volume (V_0_) which was informed by the volume of the glass of water with which IRSODFs are administered has been considered as one of the most important variables that affects the behaviors of IRSODFs [6, 9, 11, 12]. In this study, a V_0_ of 250 mL was used to compute the dose number (D_0_) of lamotrigine in adults.

#### The age-specific gastric volumes in pediatrics

Age-specific V_0_ values were used to calculate the age-specific D_0_ values for (1) neonates (birth to 27 days) [37], (2) infants (aged 6 months), and (3) infants and children of 1 year old and above [6, 11]. It is generally accepted that the solubility of a drug in biorelevant media could be affected by 2 variables: the volume and composition of the media. Earlier research has indicated that gastric pH levels in children aged 2 years and older are comparable to those found in adults [18, 38]. Contrarily, the volumes of gastric fluids vary significantly based on the age of the pediatric sub-populations. Previous studies have reported volumes of gastric fluids in the range of 0.40–0.56 mL/kg in pediatric populations [39–41]. When this range was extrapolated to an adult of 70 kg, this range corresponded to 28.0 to 37.1 mL/kg [6, 9, 11]. In fasting conditions, the volume of gastric fluids was reported in an adult of 70 kg as 40 mL [42].

To obtain the median weights of neonates, infants, and children, the growth charts developed by the Centers for Disease Control and Prevention (CDC) were used [43]. The median weights were used to calculate the age-specific V_0_ values were calculated as previously described [6]. The median weights of the different pediatric sub-populations, gastric volumes, and age-specific V_0_ values are shown in Supplementary Table S2 [9, 39–42].

Solubility values of lamotrigine in the age-specific V_0_ values in pediatric and adult populations were used to assign lamotrigine to either a high or low solubility class. If Lamotrigine’s highest dose strength is soluble in a volume equivalent to or less than the age-specific V_0_ value across various pediatric sub-populations, and over a pH range of 1.2–6.8 at 37 ± 1 °C, then it would be categorized as having a high solubility class. Otherwise, lamotrigine was said to have a low solubility class.

### Quantification of lamotrigine in the samples

Lamotrigine was determined using an HPLC-UV method as previously described in the US Pharmacopeia [44]. The mobile phase was methanol and buffer (60:40). The buffer was composed of 0.8 g/L ammonium acetate and adjusted to a pH of 4.5 using glacial acetic acid. A wavelength of 210 nm was utilized to detect lamotrigine, while the flow rate was fixed at 1 mL/min. Before use, the mobile phase was filtered using a filter with a pore size of 0.45 μm. The quantities of lamotrigine were determined against a 7-point calibration curve that was created from a methanol-based standard solution of lamotrigine in methanol in the range of 1-100 µg/mL. The solubility of lamotrigine was assessed in triplicates in each biorelevant media.

### Calculation of the dose number

Maximal dose strengths of lamotrigine were obtained from the British National Formulary (BNF) 78 (Sep 2019 to Mar 2020) [45]. Doses for pediatric populations were expressed as mg/kg. The maximal dose for each pediatric sub-population was calculated based on the median weight of that sub-population provided in the growth charts of the CDC. The age-specific D_0_ values were calculated as previously described using the age-specific V_0_, maximal dose strength, and saturated solubility [20, 22, 46].

### The physicochemical and pharmacokinetic properties of lamotrigine

The molecular descriptors and other physicochemical properties of lamotrigine like the experimentally determined water solubility and the *n*-octanol/water partition coefficient (LogP) were obtained from the DrugBank database [47]. Additionally, ALOGPS v.2.1 (The Virtual Computational Chemistry Laboratory, VCCLAB, Germany) and ChemAxon (ChemAxon, Budapest, Hungary) were used to calculate the computational *n*-octanol/water partition coefficient (cLogP). A correlation plot was created between LogP and cLogP for a set of 35 drug molecules including metoprolol that was used as a benchmark for high/low benchmark of paracellular intestinal permeability from [6, 9, 19, 21, 23, 48–50]. The correlation plot is shown in Supplementary Figure S1. ChemAxon also predicted the potential bioavailability and polar surface area of lamotrigine.

### Data analysis

The data obtained in this study were entered and handled into GraphPad Prism for Windows v.7.0. One-way analysis of variance (ANOVA) with Tukey’s multiple comparison tests was used to detect differences in the solubility of lamotrigine in the different age-specific biorelevant media. Pediatric/adult solubility ratios were computed as percentages by dividing the solubility of lamotrigine in the age-specific pediatric media by those in the equivalent adult media (µ_pediatric_/µ_adult_ × 100%). When the ratio was < 100%, this indicated inferior solubility of lamotrigine in the pediatric media compared to that in the adult media. On the other hand, when the ratio was > 100%, this indicated superior solubility of lamotrigine in the pediatric media compared to that in the adult media [11, 12]. In IVBESs, a difference of ± 20% indicated a clinically insignificant difference [12, 32, 51]. Similarly, when the mean ratios were within the 80-125% range, differences in the solubility between pediatrics and adults were considered insignificant. On the other hand, when the mean ratios were outside the 80-125% range, differences in the solubility between pediatrics and adults were considered significant.

## Results

### Solubility of lamotrigine in the age-specific biorelevant media

**Fig. 1:**
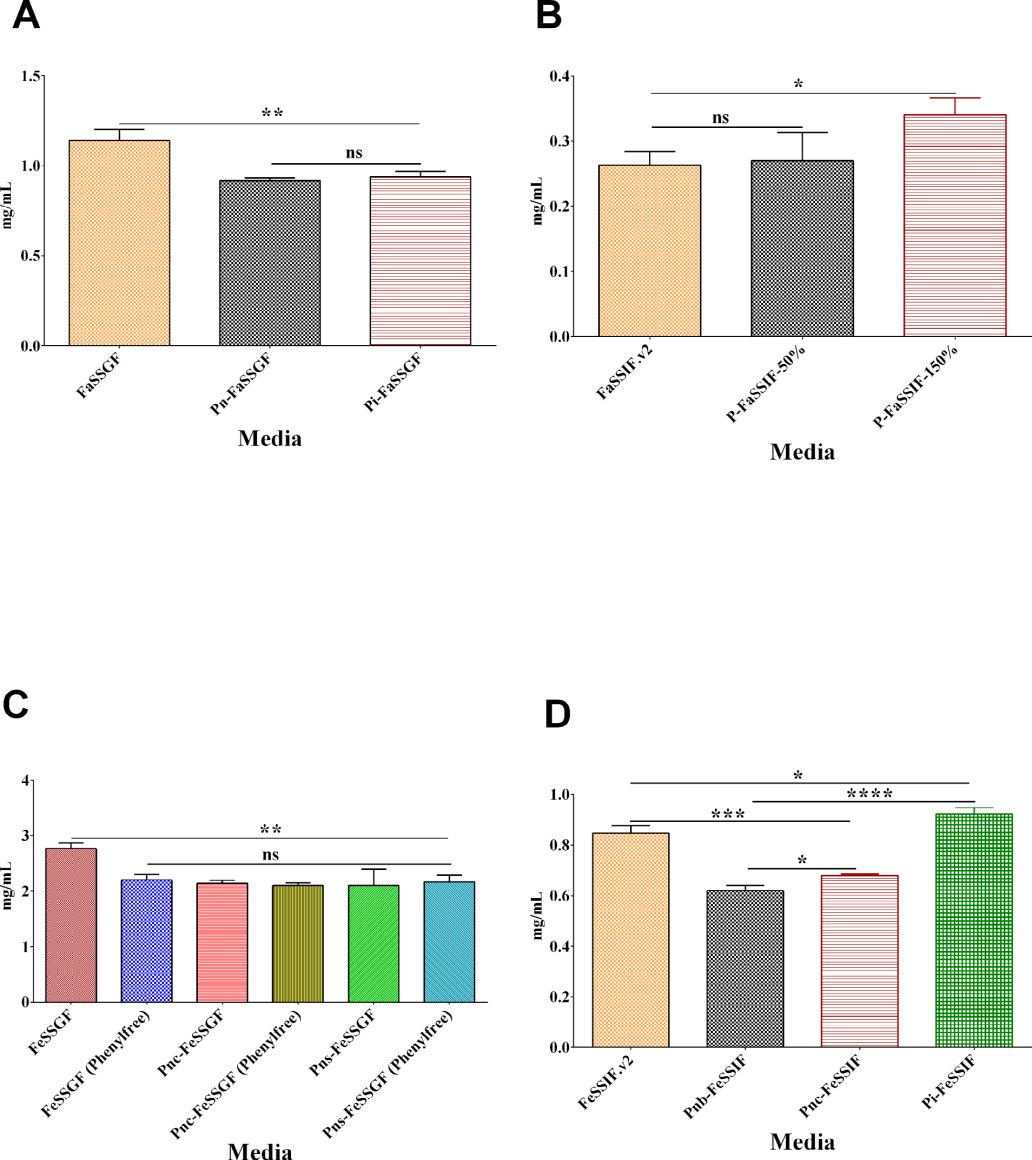
Solubility of lamotrigine in simulated (**A**) fasted-state gastric media, (**B**) fed-state gastric media, (**C**) fasted-state intestinal, and (**D**) fed-state intestinal media. Ns: not significant, *: p-value < 0.05, **: p-value < 0.01, ***: p-value < 0.001, ****: p-value < 0.0001. FaSSGF: fasted-state simulated gastric fluid; Pn-FaSSGF: pediatric fasted-state gastric media representative of neonates (birth to 27 days); Pi-FaSSGF: pediatric fasted-state gastric media representative of infants (1–12 months); FeSSGF: fed-state simulated intestinal fluid; Pnc-FeSSGF: pediatric fed-state gastric media representative of neonates (birth to 27 days)fed milk-based formula; Pns-FeSSGF: pediatric fed-state gastric media representative of neonates (birth to 27 days) fed soy-based formula; FaSSIF.v2: fasted-state simulated intestinal fluid; P-FaSSIF-50%: pediatric fasted-state intestinal media formulated with bile salt concentrations 50% (i.e., 1.5 mM) of adult levels; P-FaSSIF-150%: pediatric fasted-state intestinal media formulated with bile salt concentrations 150% (i.e., 4.5 mM) of adult levels; FeSSIF.v2: fed-state simulated gastric fluid; Pnb-FeSSIF: pediatric fed-state intestinal media representative of neonates (birth to 27 days) fed breast milk; Pnc-FeSSIF: pediatric fed-state intestinal media representative of neonates (birth to 27 days) fed milk-based formula; Pi-FeSSIF: pediatric fed-state intestinal media representative of infants (1–12 months) fed milk-based formula

Figure 1 shows the solubility of lamotrigine in the simulated gastric and intestinal media in the fasted and fed conditions of pediatric subpopulations and adults. The solubility of lamotrigine ranged from 0.90 to 1.21 mg/mL in the different simulated fasted-state gastric media (Fig. 1A), 0.24 to 0.36 mg/mL in the different simulated fasted-state intestinal media (Fig. 1B), 1.80 to 2.85 mg/mL in the different simulated fed-conditions gastric media (Fig. 1C), and 0.60 to 0.95 mg/mL in the different simulated fed-conditions intestinal media (Fig. 1D).

When compared, the solubility of lamotrigine in the simulated fasted-state gastric media of neonates and infants was significantly less than that in the simulated fasted-state gastric media of adults. On the other hand, when bile salt concentrations were increased to 150% of the adult levels in the pediatric simulated fasted-state intestinal media, the solubility of lamotrigine was significantly higher (p-value < 0.05) compared to that in fasted-state simulated intestinal fluid and pediatric fasted-state intestinal media formulated with bile salt concentrations 50% of the adult levels. Irrespective of the type of feed, the solubility of lamotrigine in fed-conditions simulated gastric fluid was found to be significantly higher (p-value < 0.01) than that in pediatric fed-conditions gastric media. Furthermore, in infant (1–12 months) fed-conditions, the solubility of lamotrigine was significantly higher (p-value < 0.001) in pediatric fed-conditions intestinal media representative of milk-based formula and fed-conditions simulated intestinal fluid than that in neonate fed-conditions intestinal media representative of breast milk and milk-based formula.

### The calculated dose numbers and solubility class of lamotrigine in the age-specific biorelevant media

According to the BCS criteria, APIs are assigned a high solubility class when their *D*_0_ values in the corresponding biorelevant media are ≤ 1. Conversely, APIs are assigned a low solubility class when their *D*_0_ values in the corresponding biorelevant media are > 1. The calculated *D*_0_ values of lamotrigine in all age-specific pediatric and adult biorelevant media. These values indicated that lamotrigine had low solubility. The calculated *D*_0_ values of lamotrigine in all age-specific biorelevant media are shown in Table 1.

**Table 1:** Age-specific dose number values in the 16 biorelevant media

Media	Dose number (D_0_)																			
	Neonate (1 day to 6 months)	Age (years)																		
		0.50	1	2	3	4	5	6	7	8	9	10	11	12	13	14	15	16	17	Adult (≥ 18)
FaSSGF	3.62	3.62	3.61	3.62	3.61	3.61	3.61	3.62	3.61	3.61	3.63	3.61	3.61	3.61	3.61	3.62	3.62	3.62	3.62	3.82
FeSSGF	1.49	1.49	1.49	1.50	1.49	1.49	1.49	1.50	1.49	1.49	1.50	1.49	1.49	1.49	1.49	1.50	1.50	1.50	1.50	1.58
FaSSIF.v2	16.57	16.57	16.55	16.57	16.55	16.56	16.57	16.57	16.56	16.56	16.63	16.53	16.54	16.54	16.53	16.60	16.59	16.58	16.58	17.50
FeSSIF.v2	4.52	4.52	4.51	4.52	4.51	4.52	4.52	4.52	4.52	4.52	4.54	4.51	4.51	4.51	4.51	4.53	4.52	4.52	4.52	4.77
Pn-FaSSGF	4.85	4.85	4.84	4.85	4.84	4.85	4.85	4.85	4.85	4.85	4.87	4.84	4.84	4.84	4.84	4.86	4.85	4.85	4.85	5.12
Pi-FaSSGF	4.73	4.73	4.73	4.73	4.73	4.73	4.73	4.73	4.73	4.73	4.75	4.72	4.73	4.73	4.72	4.74	4.74	4.74	4.74	5.00
Pnc-FeSSGF	2.09	2.09	2.09	2.09	2.09	2.09	2.09	2.09	2.09	2.09	2.10	2.09	2.09	2.09	2.09	2.10	2.10	2.09	2.09	2.21
Pns-FeSSGF	1.89	1.89	1.89	1.89	1.89	1.89	1.89	1.89	1.89	1.89	1.90	1.89	1.89	1.89	1.89	1.90	1.90	1.89	1.90	2.00
P-FaSSIF-50%	14.73	14.73	14.71	14.73	14.71	14.72	14.72	14.73	14.72	14.72	14.78	14.69	14.71	14.71	14.70	14.76	14.74	14.73	14.74	15.56
P-FaSSIF-150%	11.36	11.36	11.35	11.36	11.35	11.35	11.36	11.36	11.36	11.36	11.40	11.33	11.34	11.34	11.34	11.38	11.37	11.37	11.37	12.00
Pnb-FeSSIF	6.63	6.63	6.62	6.63	6.62	6.62	6.63	6.63	6.62	6.63	6.65	6.61	6.62	6.62	6.61	6.64	6.64	6.63	6.63	7.00
Pnc-FeSSIF	5.85	5.85	5.84	5.85	5.84	5.84	5.85	5.85	5.85	5.85	5.87	5.83	5.84	5.84	5.84	5.86	5.85	5.85	5.85	6.18
Pi-FeSSIF	4.19	4.19	4.18	4.19	4.18	4.18	4.18	4.19	4.18	4.18	4.20	4.18	4.18	4.18	4.18	4.19	4.19	4.19	4.19	4.42
FeSSGF (Phenylfree)	1.37	1.37	1.37	1.37	1.37	1.37	1.37	1.37	1.37	1.37	1.38	1.37	1.37	1.37	1.37	1.37	1.37	1.37	1.37	1.45
Pnc-FeSSGF (Phenylfree)	1.42	1.42	1.42	1.42	1.42	1.42	1.42	1.42	1.42	1.42	1.43	1.42	1.42	1.42	1.42	1.42	1.42	1.42	1.42	1.50
Pns-FeSSGF (Phenylfree)	1.41	1.41	1.41	1.41	1.41	1.41	1.41	1.41	1.41	1.41	1.42	1.41	1.41	1.41	1.41	1.41	1.41	1.41	1.41	1.49

### Pediatric to adult solubility ratios of lamotrigine

**Fig. 2:**
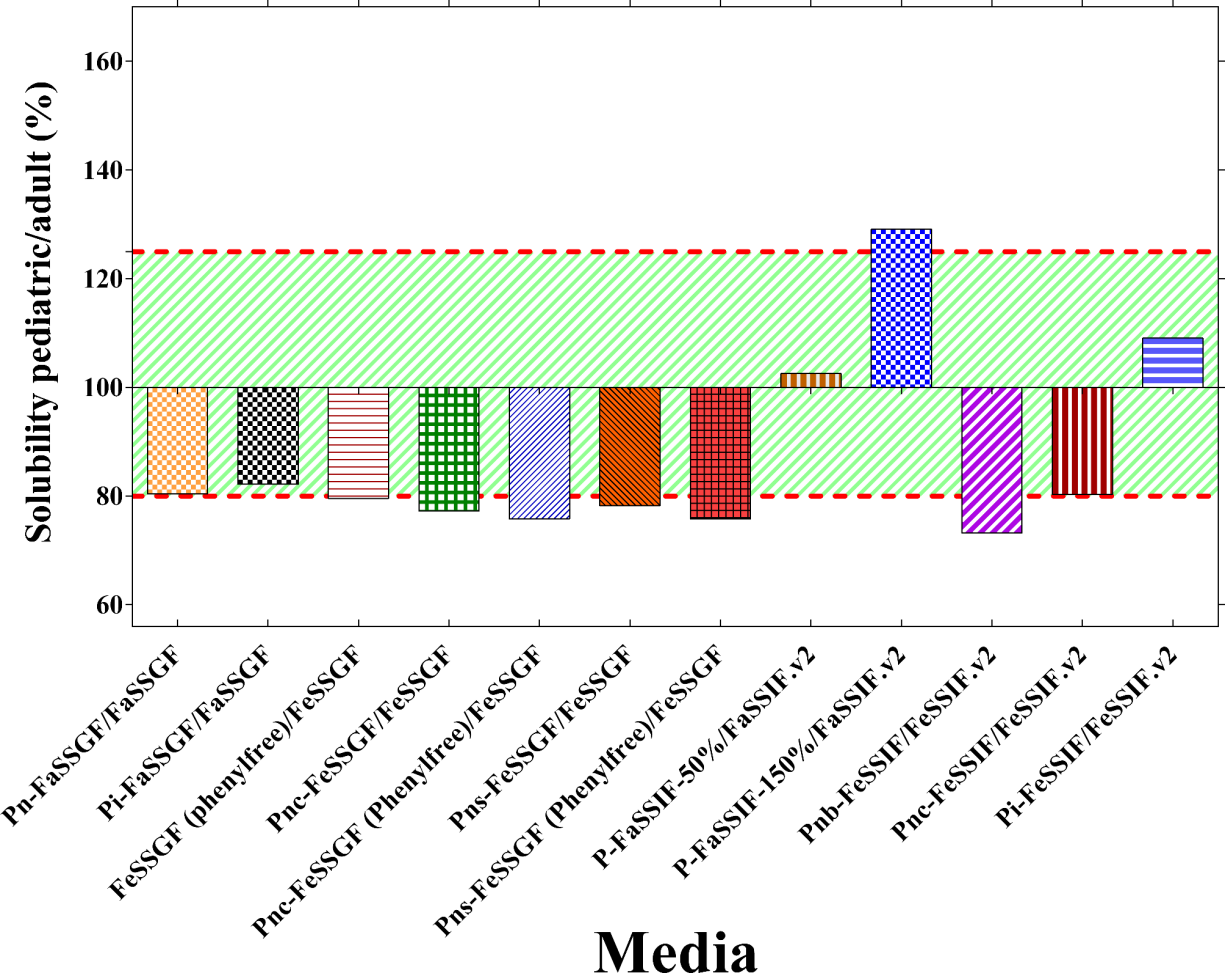
Age-specific pediatric/adult solubility ratios

The pediatric/adult solubility ratios of lamotrigine are shown in Fig. 2. The pediatric/adult solubility ratios of lamotrigine fell outside the 80-125% range in 6 (50.0%) and were borderline in 3 (25.0%) out of 12 compared media. These ratios indicated that the solubility of lamotrigine showed considerable differences in 9 out of the 12 (75.0%) of the compared media.

### Permeability class

The experimentally determined LogP of lamotrigine was 1.93. ALOGPS predicted a cLoP of 1.87 and ChemAxon predicted a cLogP of 1.93 for lamotrigine. The polar surface area of lamotrigine as predicted by ChemAxon was 90.71 Å^2^. Metoprolol was previously used as a benchmark for low/high intestinal permeability. The experimentally determined LogP was 2.15 and the polar surface area of metoprolol was 50.72 Å^2^. ALOGPS predicted a cLogP of 1.80 and ChemAxon predicted a cLogP of 1.76 for metoprolol. Lamotrigine cannot be conclusively assigned to either a high or a low permeability class. Previous studies classified APIs to a low permeability class when they had LogP values higher than that of metoprolol. Similarly, APIs were assigned to a low permeability class when they had a polar surface area of less than 90.0 Å^2^.

Previous studies reported conflicting bioavailability values for lamotrigine in humans. While some studies reported bioavailability values approaching 100% [52, 53], other studies reported lower absolute bioavailability values of 73% ± 16% [54]. The absorption of lamotrigine was reported to be unaffected by food and the drug did not undergo first-pass metabolism [55]. The geometric mean of time to maximal peak drug concentration was 1.43 (95% CI: 0.00–4.00) hours [54]. Therefore, lamotrigine could be assigned either a low or high permeability class.

Compared to metoprolol which was previously used as a benchmark for low/high intestinal permeability, metoprolol had an experimentally determined LogP of 2.15 and a polar surface area of 50.72 Å^2^. This indicated that lamotrigine had low permeability. Experimentally determined LogP values of 36 drugs were plotted against their corresponding cLogP showed excellent correlation (Supplementary Figure S1). The molecular descriptors of lamotrigine are shown in Supplementary Table S3.

## Discussion

In clinical practice, the use of drugs for unlicensed or off-label objectives has been reported among pediatric populations. Off-label use encompasses the use of drugs for an unapproved indication, in an unapproved patient population, in unapproved dosages or regimens, *via* unapproved routes of administration, in unapproved combinations, and/or with unapproved modified formulations. The use of off-label drugs has limited efficacy and safety. Additionally, such use can cause significant harm or toxicity to these fragile patients. Therefore, it is essential to evaluate the behaviors of traditional, re-formulated, modified, and new dosage forms in pediatric as well as adult populations before they can be used. For the first time, the solubility of lamotrigine was evaluated in age-specific biorelevant media that simulated fasted- and fed-conditions gastric and intestinal environments. In this study, the impact of the developmental changes in volume and composition of the gastrointestinal fluids on the solubility and subsequently the behavior of IRSODFs containing lamotrigine were assessed. The findings of this study could be informative to formulation scientists, pediatricians, neurologists, and epileptologists who could be interested in optimizing the pharmacotherapy of epilepsy in pediatric populations.

In this study, we assessed the solubility of lamotrigine in simulated age-specific biorelevant media. It is widely accepted that pediatric patients should not be considered miniatures of their adult peers. Therefore, age-specific biopharmaceutical criteria should be applied to the different pediatric sub-populations [6, 11, 56]. In ideal conditions, luminal fluids that could be aspirated from the human gastrointestinal tract of pediatric patients that belong to different age groups might be used to assess the solubility of lamotrigine ex vivo. However, aspiration of luminal fluids can be hampered by many ethical and logistic issues, notably in pediatric patients [6, 11]. Therefore, the age-specific biorelevant media that were used in this study simulated the unique intricacies of the environments in the gastric and proximal small intestines of adults and pediatric sub-populations [11, 12, 32]. While developing these age-specific biorelevant media, to imitate the in vivo conditions, the levels of phospholipids, byproducts of fat digestion, pepsin, and osmolality were estimated [12, 32]. The age-appropriateness of the simulated biorelevant media was previously demonstrated for many drugs [11, 12, 57, 58].

The biorelevant media used in this study accounted for the different types of feed including breast milk, cow milk-based formulas, soy-based formulas, and a phenylalanine-free formula. This should have ensured assessing the potential effects of these feed types on the solubility of lamotrigine. For the first time, a phenylalanine-free formula was added in this study. This should have extended the findings reported in this study to pediatric patients with phenylketonuria who could be fed phenylalanine-free formulas. The composition and quantity of human milk samples can vary significantly within and between donors [12, 59, 60]. Additionally, it is difficult to obtain human breast milk samples and maintain their uniformity. Therefore, the use of human breast milk samples obtained from lactating women to prepare biorelevant media has been discouraged due to these logistic and technical difficulties. On the other hand, nutritional companies are required to conduct quality control tests to ensure the between batches uniformity of the manufactured infant formulas [61]. As formulas are commonly used as substitutes for human breast milk, they are designed to mirror the concentrations of carbohydrates, proteins, and fats in human breast milk [61, 62]. In this study, the formulas were used to ensure that the prepared biorelevant media were reproducible. Bile salts could modulate the solubility of some compounds [63]. It is widely accepted that pediatric biopharmaceutics is still inconclusive and previous studies have reported significant variability in the concentrations of bile salts between pediatric and adult populations [12, 32, 63]. Therefore, pharmaceutical scientists accounted for these reported variabilities by developing media using 150% and 50% of the adult concentrations [12]. There was no need to develop a media with similar (100%) concentrations in adults because this was reflected in the media that corresponded to the adults. Because data relevant to buffering capacity, osmolality, and phospholipids in the intestinal fluids of pediatrics were not available, pediatric media were developed maintaining the same bile salt/lecithin ratio as in the adults [11, 12].

In this study, lamotrigine had low solubility as indicated by the calculated D_0_ values in the different biorelevant media that was calculated based on customized V_0_ values for each age group. A similar low solubility of lamotrigine was reported in some pediatric and adult biorelevant media [29]. This means that lamotrigine would exhibit solubility-limited absorption, notably in the small intestine where the majority of absorption would occur. Solubility-limited absorption and first-pass effect are frequent causes of low systemic bioavalability of orally administered drug molecules [1, 10]. Drug molecules with low solubility in the gastrointestinal fluids (BCS 2 and 4 drugs) are more likely to exhibit erratic or variable bioavailability. Therefore, assessing solubility of drug molecules in biorelevant media can be used to predict the biopharmaceutical performance of traditional, re-formulated, and novel IRSODFs [64]. In this study, the solubility of lamotrigine was evaluated in different biorelevant media that represented the fasted and fed conditions of the gastrointestinal tract, notably, the stomach and proximal small intestine in this study. The results showed noteworthy variations in the solubility measurements of lamotrigine in biorelevant media specific to each age group. These differences predicted notable differences in the biopharmaceutical performance of IRSODFs containing lamotrigine that are intended for oral administration. These findings of this study indicated that lamotrigine might exhibit erratic or variable bioavailability in different pediatric sub-populations. Fluctuations in the serum concentration of antiseizure medications was shown to be associated with higher frequency of seizures and/or side effects [65, 66]. The results presented in this study were in line with those reported in previous research in which the BCS solubility class of phenobarbital, diazepam, celecoxib, cephalexin, doxycycline, amoxicillin, erythromycin, chloramphenicol, trimethoprim, and prednisolone changed from high to low in some pediatric sub-populations [6, 11, 29]. Despite the differences in the solubility values, lamotrigine exhibited low solubility in all age-specific adult and pediatric biorelevant media. Additionally, the pediatric-to-adult solubility ratios fell outside or were borderline the 80-125% in the majority (75%) of the biorelevant media compared. These findings indicated considerable variabilities in the performance of IRSODFs containing lamotrigine. These might be translated as failures to demonstrate bioequivalence of generic IRSODFs compared to innovator drugs. In previous studies, pediatric to adult solubility ratios were outside or borderline the 80-125% in different biorelevant media for several low solubility drug molecules including carbamazepine, phenytoin, dapsone, griseofulvin, indomethacin, spironolactone, fenofibrate, and celecoxib [11, 12].

The data pertinent to the intestinal permeability of lamotrigine did not permit a conclusive assignment of lamotrigine to either the high or low permeability class. Lamotrigine is a small molecule with a molecular weight smaller than that of metoprolol that is often used as a benchmark of low/high intestinal permeability (256.091 g/mol vs. 267.364 g/mol) [6, 9, 19, 21–23, 48]. Additionally, lamotrigine was reportedly to have nearly complete absorption as demonstrated by an absolute bioavailability of nearly 100%. Studies using Caco-2 showed that lamotrigine had higher pearmability compared to metoprolol. The apical to basolateral apparent permeability was 73.7 ± 8.7 × 10^− 6^ cm/s and the basolateral to apical apparent permeability was 41.4 ± 1.6 × 10^− 6^ cm/s [31]. Moreover, lamotrigine crosses the blood-brain barrier and achieves therapeutic concentrations in the brain. These can support assigning lamotrigine to a high permeability class. Contrarily, lamotrigine exhibits less lipophilicity compared to metoprolol as indicated by the experimentally determined LogP values for both drugs. Additionally, lamotrigine has a larger polar surface area than metoprolol which can preclude lamotrigine from permeating through the intestinal wall. Moreover, some studies reported less than complete absorption of lamotrigine. These, on the other hand, support assigning lamotrigine to a low permeability class. Despite the lack of availability of pediatric biopharmaceutical data, permeability kinetics in 2 years and older children are considered similar to those in adults [9, 18]. However, higher paracellular permeability is often expected in younger pediatric sub-populations. Solely based on the paracellular permeability data, lamotrigine could be assigned a low permeability class. However, more data are still needed to draw a more solid conclusion on the impact of the higher paracellular permeability in younger pediatric sub-populations on the permeability class of lamotrigine. Given the low solubility and potentially low permeability of lamotrigine, greater variability could be expected in the performance of IRSODFs in younger pediatric sub-populations.

### Limitations of the study

The main limitation of this study is that the age-specific media were adopted and modified from the literature [11, 12]. It is noteworthy mentioning that this is the first assessment of the solubility of lamotrigine in age-specific biorelevant media. In clinical practice, lamotrigine is commonly prescribed for adults and different pediatric sub-populations. Today, different IRSODFs containing lamotrigine are available. These oral formulations include orally disintegrating tablets. These tables can be either swallowed whole with a glass of water, chewed, or dispersed in water. These dosage forms could be suitable for patients who have difficulties swallowing IRSODFs. As a result, the outcomes of this study hold practical significance for pediatricians, neurologists, epileptologists, and formulation scientists. The FDA approved the use of lamotrigine in children who are 2 years and older. Nonetheless, the solubility of lamotrigine was evaluated in biorelevant media intended to simulate fasted and fed states in neonates. Additionally, differences in solubility of lamotrigine in pediatric sub-populations and adults were assessed based on the ratio boundaries (80-125%) used to establish bioequivalence between originator/reference listed drugs and their generic counterparts [12, 32, 51]. In this study, the changes in the solubility of lamotrigine were used to assess the performance of IRSODFs. It is noteworthy mentioning that the performance of IRSODFs could be influenced by other variables like gastric and intestinal motility, residence time, pre-systemic metabolism, degradation of the drug before absorption, intestinal permeability, and presence of transport systems in the gastrointestinal tract [1, 10, 64, 67]. Therefore, physiologically-based pharmacokinetic models that would comprehensively take all these variables into account might be used to more reliably predict the performance of IRSODFs. As per the present BCS criteria, in vitro dissolution testing would not be advisable for establishing interchangeability or similarity between IRSODFs containing lamotrigine as an API in adults or pediatric sub-populations, regardless of whether they are traditional, re-formulated, or novel. However, IVBESs would be still needed to demonstrate bioequivalence, interchangeability, or similarity between the reference listed drug product and traditional, re-formulated, and novel IRSODFs that contain lamotrigine as an API for use in adults or pediatric sub-populations. Using the BCS principles, the biowaivers special interest group of the International Pharmaceutical Federation provides recommendations for or against the use of in vitro dissolution studies in place of IVBESs (biowaiver) using the flow diagram shown in Supplementary Fig. 2.

## Conclusion

The findings of this study demonstrated that lamotrigine has low solubility in the age-specific biorelevant media for adults and different pediatric sub-populations. The study also showed significant differences in the solubility of lamotrigine in different age-specific biorelevant media and IRSODFs containing lamotrigine could exhibit significant variability in their in vivo performance. Therefore, the current BCS-based criteria might not be directly applied in pediatric sub-populations. Future studies are still needed to generate more pediatric biopharmaceutical data to help understand the performance of IRSODFs in pediatric sub-populations.

### Electronic supplementary material

Below is the link to the electronic supplementary material.

Supplementary Material 1

## Data Availability

All data relevant to this study were included in the results section of this manuscript. Additional data are provided as supplementary materials.

## References

[1] Amidon GL, Lennernas H, Shah VP, Crison JR (1995). A theoretical basis for a biopharmaceutic drug classification: the correlation of in vitro drug product dissolution and in vivo bioavailability. Pharm Res.

[2] (EMA) EMA. : Committee for Medicinal Products for Human Use. Guideline on the investigation of bioequivalence. In.; 2010.

[3] (FDA) UFaDA. : Draft Guidance. Guidance for industry: waiver of in vivo bioavailability and bioequivalence studies for immediate-release solid oral dosage forms based on a biopharmaceutics classification system. In: *Food and Drug Administration, Rockville, MD* 2015.

[4] Kortejärvi H, Malkki J, Shawahna R, Scherrmann JM, Urtti A, Yliperttula M (2014). Pharmacokinetic simulations to explore dissolution criteria of BCS I and III biowaivers with and without MDR-1 efflux transporter. Eur J Pharm Sciences: Official J Eur Federation Pharm Sci.

[5] Kortejärvi H, Shawahna R, Koski A, Malkki J, Ojala K, Yliperttula M (2010). Very rapid dissolution is not needed to guarantee bioequivalence for biopharmaceutics classification system (BCS) I drugs. J Pharm Sci.

[6] Shawahna R (2016). Pediatric Biopharmaceutical classification system: using age-appropriate initial gastric volume. AAPS J.

[7] Polli JE (2008). In vitro studies are sometimes better than conventional human pharmacokinetic in vivo studies in assessing bioequivalence of immediate-release solid oral dosage forms. AAPS J.

[8] Abdel-Rahman SM, Amidon GL, Kaul A, Lukacova V, Vinks AA, Knipp GT (2012). Summary of the National Institute of Child Health and Human Development-best pharmaceuticals for Children Act Pediatric Formulation Initiatives Workshop-Pediatric Biopharmaceutics classification system Working Group. Clin Ther.

[9] Batchelor H (2014). Paediatric biopharmaceutics classification system: current status and future decisions. Int J Pharm.

[10] Batchelor HK, Fotaki N, Klein S (2014). Paediatric oral biopharmaceutics: key considerations and current challenges. Adv Drug Deliv Rev.

[11] Shawahna R, Zyoud A, Haj-Yahia A, Taya R (2021). Evaluating solubility of Celecoxib in Age-Appropriate fasted- and Fed-State gastric and intestinal Biorelevant Media Representative of Adult and Pediatric Patients: implications on Future Pediatric Biopharmaceutical classification system. AAPS PharmSciTech.

[12] Maharaj AR, Edginton AN, Fotaki N (2016). Assessment of Age-Related changes in Pediatric Gastrointestinal solubility. Pharm Res.

[13] Martir J, Flanagan T, Mann J, Fotaki N (2020). BCS-based biowaivers: extension to paediatrics. Eur J Pharm Sciences: Official J Eur Federation Pharm Sci.

[14] Bhatt-Mehta V, Hammoud H, Amidon GL (2020). A proposed pediatric biopharmaceutical classification system for medications for chronic diseases in children. Eur J Pharm Sciences: Official J Eur Federation Pharm Sci.

[15] Elder DP, Holm R, Kuentz M (2017). Medicines for Pediatric Patients-Biopharmaceutical, Developmental, and Regulatory Considerations. J Pharm Sci.

[16] Del Moral Sanchez JM, Gonzalez-Alvarez I. Biopharmaceutical optimization in neglected diseases for paediatric patients by applying the provisional paediatric biopharmaceutical classification system. 2018, 84(10):2231–41. 10.1111/bcp.13650 PMC6138508 29846973

[17] Gandhi SV, Rodriguez W, Khan M, Polli JE (2014). Considerations for a Pediatric Biopharmaceutics classification system (BCS): application to five drugs. AAPS PharmSciTech.

[18] Batchelor HK, Kendall R, Desset-Brethes S, Alex R, Ernest TB (2013). Application of in vitro biopharmaceutical methods in development of immediate release oral dosage forms intended for paediatric patients. Eur J Pharm Biopharmaceutics: Official J Arbeitsgemeinschaft fur Pharmazeutische Verfahrenstechnik eV.

[19] Dahan A, Wolk O, Kim YH, Ramachandran C, Crippen GM, Takagi T, Bermejo M, Amidon GL (2013). Purely in silico BCS classification: Science based quality standards for the world’s drugs. Mol Pharm.

[20] Wolk O, Agbaria R, Dahan A (2014). Provisional in-silico biopharmaceutics classification (BCS) to guide oral drug product development. Drug Des Devel Ther.

[21] Kasim NA, Whitehouse M, Ramachandran C, Bermejo M, Lennernäs H, Hussain AS, Junginger HE, Stavchansky SA, Midha KK, Shah VP (2004). Molecular properties of WHO essential drugs and provisional biopharmaceutical classification. Mol Pharm.

[22] Lindenberg M, Kopp S, Dressman JB (2004). Classification of orally administered drugs on the World Health Organization Model list of essential Medicines according to the biopharmaceutics classification system. Eur J Pharm Biopharmaceutics: Official J Arbeitsgemeinschaft fur Pharmazeutische Verfahrenstechnik eV.

[23] Shawahna R, Rahman N (2011). Evaluation of the use of partition coefficients and molecular surface properties as predictors of drug absorption: a provisional biopharmaceutical classification of the list of national essential medicines of Pakistan. Daru: J Fac Pharm Tehran Univ Med Sci.

[24] Charoo NA, Cristofoletti R, Dressman JB (2015). Risk assessment for extending the Biopharmaceutics classification system-based biowaiver of immediate release dosage forms of fluconazole in adults to the paediatric population. J Pharm Pharmacol.

[25] Brigo F, Igwe SC, Lattanzi S (2019). Ethosuximide, sodium valproate or lamotrigine for absence seizures in children and adolescents. Cochrane Database Syst Rev.

[26] Brigo F, Jones K, Eltze C, Matricardi S (2021). Anti-seizure medications for Lennox-Gastaut syndrome. Cochrane Database Syst Rev.

[27] Besag FMC, Vasey MJ, Sharma AN, Lam ICH (2021). Efficacy and safety of lamotrigine in the treatment of bipolar disorder across the lifespan: a systematic review. Ther Adv Psychopharmacol.

[28] Bendtsen L, Zakrzewska JM, Abbott J, Braschinsky M, Di Stefano G, Donnet A, Eide PK, Leal PRL, Maarbjerg S, May A (2019). European Academy of Neurology guideline on trigeminal neuralgia. Eur J Neurol.

[29] Caleffi-Marchesini ER, Borghi-Pangoni FB, Macente J, Chiamulera-Mantovani P, Mazucheli J, Cristofoletti R, Diniz A (2023). Exploring in vitro solubility of lamotrigine in physiologically mimetic conditions to prospect the in vivo dissolution in pediatric population. Biopharm Drug Dispos.

[30] Porat D, Azran C, Mualem Y, Vainer E, Gibori R, Vaynshtein J, Dukhno O, Dahan A (2022). Lamotrigine therapy in patients after bariatric surgery: potentially hampered solubility and dissolution. Int J Pharm.

[31] Vaithianathan S, Raman S, Jiang W, Ting TY, Kane MA, Polli JE (2015). Biopharmaceutic Risk Assessment of brand and generic lamotrigine tablets. Mol Pharm.

[32] Jantratid E, Janssen N, Reppas C, Dressman JB (2008). Dissolution media simulating conditions in the proximal human gastrointestinal tract: an update. Pharm Res.

[33] Juenemann D, Bohets H, Ozdemir M, de Maesschalck R, Vanhoutte K, Peeters K, Nagels L, Dressman JB (2011). Online monitoring of dissolution tests using dedicated potentiometric sensors in biorelevant media. Eur J Pharm Biopharmaceutics: Official J Arbeitsgemeinschaft fur Pharmazeutische Verfahrenstechnik eV.

[34] (WHO) WHO. : Proposal to waive in vivo bioequivalence requirements for WHO Model List of Essential Medicines immediate-release, solid oral dosage forms. In.: Technical Report Series; 2006.

[35] Davit BM, Kanfer I, Tsang YC, Cardot J-M (2016). BCS biowaivers: similarities and differences among EMA, FDA, and WHO requirements. AAPS J.

[36] Preparations, WECoSfP. WHO Technical Report Series, No. 937, Annex 8. Proposal to waive in vivo bioequivalence requirements for WHO Model List of Essential Medicines immediate-release, solid oral dosage forms. *World Health Organization: Geneva* 2006.

[37] Williams K, Thomson D, Seto I, Contopoulos-Ioannidis DG, Ioannidis JP, Curtis S, Constantin E, Batmanabane G, Hartling L, Klassen T (2012). Standard 6: age groups for pediatric trials. Pediatrics.

[38] Bartelink IH, Rademaker CM, Schobben AF, van den Anker JN (2006). Guidelines on paediatric dosing on the basis of developmental physiology and pharmacokinetic considerations. Clin Pharmacokinet.

[39] Meakin G, Dingwall A, Addison G (1987). Effects of fasting and oral premedication on the pH and volume of gastric aspirate in children. Br J Anaesth.

[40] Schwartz DA, Connelly NR, Theroux CA, Gibson CS, Ostrom DN, Dunn SM, Hirsch BZ, Angelides AG (1998). Gastric contents in children presenting for upper endoscopy. Anesth Analg.

[41] Crawford M, Lerman J, Christensen S, Farrow-Gillespie A (1990). Effects of duration of fasting on gastric fluid pH and volume in healthy children. Anesth Analg.

[42] Goetze O, Treier R, Fox M, Steingoetter A, Fried M, Boesiger P, Schwizer W (2009). The effect of gastric secretion on gastric physiology and emptying in the fasted and fed state assessed by magnetic resonance imaging. Neurogastroenterol Motil.

[43] (CDC) CfDCaP. : Stature-for-age and weight-for-age percentiles. 2 to 20 years: boys. In.; 2000.

[44] Pharmacopeia US. Lamotrigine Monograph. In. (Rockville, MD); 2014.

[45] Baxter K, Aikman K, Luckhurst R. British National Formulary 78 (BNF). Royal Phamaceutical Society 2019:1–1701.

[46] Oh DM, Curl RL, Amidon GL (1993). Estimating the fraction dose absorbed from suspensions of poorly soluble compounds in humans: a mathematical model. Pharm Res.

[47] Celecoxib. [https://go.drugbank.com/drugs/DB00482].

[48] Wang T, Zhao P, Zhao Q, Wang B, Wang S (2016). The mechanism for increasing the oral bioavailability of poorly water-soluble drugs using uniform mesoporous carbon spheres as a carrier. Drug Delivery.

[49] Kortejarvi H, Malkki J, Shawahna R, Scherrmann JM, Urtti A, Yliperttula M (2014). Pharmacokinetic simulations to explore dissolution criteria of BCS I and III biowaivers with and without MDR-1 efflux transporter. Eur J Pharm Sciences: Official J Eur Federation Pharm Sci.

[50] Kortejarvi H, Shawahna R, Koski A, Malkki J, Ojala K, Yliperttula M (2010). Very rapid dissolution is not needed to guarantee bioequivalence for biopharmaceutics classification system (BCS) I drugs. J Pharm Sci.

[51] Guidance F. Guidance for industry. Bioavailability and bioequivalence studies submitted in NDAs or INDs—general considerations. In.; 2019.

[52] Tompson DJ, Ali I, Oliver-Willwong R, Job S, Zhu L, Lemme F, Hammer AE, Vuong A, Messenheimer JA (2008). Steady-state pharmacokinetics of lamotrigine when converting from a twice-daily immediate-release to a once-daily extended-release formulation in subjects with epilepsy (the COMPASS study). Epilepsia.

[53] Evangelista S (2005). Talnetant GlaxoSmithKline. Curr Opin Invest Drugs (London England: 2000).

[54] Polepally AR, Remmel RP, Brundage RC, Leppik IE, Rarick JO, Ramsay RE, Birnbaum AK (2015). Steady-state pharmacokinetics and bioavailability of immediate-release and extended-release formulations of lamotrigine in elderly epilepsy patients: use of stable isotope methodology. J Clin Pharmacol.

[55] Garnett WR (1997). Lamotrigine: pharmacokinetics. J Child Neurol.

[56] Kaye JL (2011). Review of paediatric gastrointestinal physiology data relevant to oral drug delivery. Int J Clin Pharm.

[57] Clarysse S, Brouwers J, Tack J, Annaert P, Augustijns P (2011). Intestinal drug solubility estimation based on simulated intestinal fluids: comparison with solubility in human intestinal fluids. Eur J Pharm Sciences: Official J Eur Federation Pharm Sci.

[58] Augustijns P, Wuyts B, Hens B, Annaert P, Butler J, Brouwers J (2014). A review of drug solubility in human intestinal fluids: implications for the prediction of oral absorption. Eur J Pharm Sciences: Official J Eur Federation Pharm Sci.

[59] Hibberd CM, Brooke OG, Carter ND, Haug M, Harzer G (1982). Variation in the composition of breast milk during the first 5 weeks of lactation: implications for the feeding of preterm infants. Arch Dis Child.

[60] Martin CR, Ling PR, Blackburn GL. Review of infant feeding: key features of breast milk and infant formula. Nutrients 2016, 8(5). 10.3390/nu8050279 PMC4882692 27187450

[61] Wargo WF (2016). The history of Infant Formula: Quality, Safety, and standard methods. J AOAC Int.

[62] Delplanque B, Gibson R, Koletzko B, Lapillonne A, Strandvik B (2015). Lipid quality in Infant Nutrition: current knowledge and Future Opportunities. J Pediatr Gastroenterol Nutr.

[63] Mithani SD, Bakatselou V, TenHoor CN, Dressman JB (1996). Estimation of the increase in solubility of drugs as a function of bile salt concentration. Pharm Res.

[64] Fotaki ARMANEN. Assessment of Age-Related changes in Pediatric Gastrointestinal solubility. Pharm Res 2015. 10.1007/s11095-015-1762-7 26220249

[65] Kilpatrick ES, Forrest G, Brodie MJ (1996). Concentration–effect and concentration–toxicity relations with lamotrigine: a prospective study. Epilepsia.

[66] Frank LM, Enlow T, Holmes GL, Manasco P, Concannon S, Chen C, Womble G, Casale EJ (1999). Lamictal (lamotrigine) monotherapy for typical absence seizures in children. Epilepsia.

[67] Zhangb S-Y, Liua C-X, Nai-Ning Songa b (2004). Overview of factors affecting oral drug absorption. Asian J Drug Metabolism Pharmacokinet.

