# Supplementary material for: Solubility of lamotrigine in age-specific biorelevant media that simulated the fasted- and fed-conditions of the gastric and intestinal environments in pediatrics and adults: implications for traditional, re-formulated, modified, and new oral formulations
Source: BMC Biotechnol. 2023 Sep 8;23:36. doi: 10.1186/s12896-023-00809-2 (PMC10492362; doi:10.1186/s12896-023-00809-2)
Supplement: Supplementary file 1 — Supplementary Material 1 [file 12896_2023_809_MOESM1_ESM.docx]

**Supplementary materials for the manuscript:**

**Solubility of lamotrigine in age-specific biorelevant media that simulated the fasted- and fed-conditions of the gastric and intestinal environments in pediatrics and adults: implications for traditional, re-formulated, modified, and new oral formulations**

Ramzi Shawahna^1,2*^, Hala Saba'aneh^3^, Amal Daraghmeh^3^, Yara Qassarwi^3^, Valentina Franco^4,5^, Xavier Declèves^6,7^

^1^Department of Physiology, Pharmacology and Toxicology, Faculty of Medicine and Health Sciences, An-Najah National University, Nablus, Palestine

^2^Clinical Research Center, An-Najah National University Hospital, Nablus, 44839, Palestine

^3^Department of Pharmacy, Faculty of Medicine and Health Sciences, An-Najah National University, Nablus, Palestine

^4^Section of Translational Neurovascular Research, IRCCS Mondino Foundation, Via Mondino 2, 27100 Pavia, Italy

^5^Clinical and Experimental Pharmacology Unit, Department of Internal Medicine and Therapeutics, University of Pavia, 27100, Pavia, Italy

^6^Biologie du Médicament-Toxicologie, AP-HP, Hôpital Cochin, 27 rue du Faubourg St. Jacques, 75679 Paris, France.

^7^Faculty of Health, Université Paris Cité, Inserm, UMRS-1144, Optimisation Thérapeutique en Neuropsychopharmacologie, 75006 Paris, France.

**^*^Correspondence:**

Ramzi Shawahna, PhD, Department of Physiology, Pharmacology and Toxicology, Faculty of Medicine & Health Sciences, New Campus, Building: 19, Office: 1340, An-Najah National University, P.O. Box 7, Nablus, Palestine

Phone: + (970) 923 45113 ext 2772

Phone: + (970) 92349739

Email: [ramzi_shawahna@hotmail.com](mailto:ramzi_shawahna@hotmail.com)

**Supplementary Table S1:** The recipe used to prepare the 16 age-specific biorelevant media. Detailed preparation and characterization methods were described previously [[1](#_ENREF_1), [2](#_ENREF_2)]

|  | **1** | **2** | **3** | **4** | **5** | **6** | **7** | **8** | **9** | **10** | **11** | **12** | **13** | **14** | **15** | **16** |
| --- | --- | --- | --- | --- | --- | --- | --- | --- | --- | --- | --- | --- | --- | --- | --- | --- |
| **Component** | **FaSSGF** | **Pn-FaSSGF** | **Pi-FaSSGF** | **FeSSGF** | **FeSSGF (Phenylfree)** | **Pnc-FeSSGF** | **Pnc-FeSSGF (Phenylfree)** | **Pns-FeSSGF** | **Pns-FeSSGF (Phenylfree)** | **FaSSIF.v2** | **P-FaSSIF-50%** | **P-FaSSIF-150%** | **FeSSIF.v2** | **Pnb-FeSSIF** | **Pnc-FeSSIF** | **Pi-FeSSIF** |
| Sodium taurocholate | 80 (uM) | 20 (uM) | 60 (uM) | – | – | – | – | – | – | 3 (mM) | 1.5 (mM) | 4.5 (mM) | 10 (mM) | 2.5 (mM) | 2.5 (mM) | 7.5 (mM) |
| Lecithin | 20 (uM) | 5 (uM) | 15 (uM) | – | – | – | – | – | – | 0.2 (mM) | 0.1 (mM) | 0.3 (mM) | 2 (mM) | 0.5 (mM) | 0.5 (mM) | 1.5 (mM) |
| Pepsin (mg/mL) | 0.1 | 0.015 | 0.025 | – | – | – | – | – | – | – | – | – | – | – | – | – |
| Sodium chloride (mM) | 34.2 | 34.2 | 34.2 | 237.02 | 237.02 | 100.35 | 100.35 | 94.79 | 94.79 | 68.62 | 68.62 | 68.62 | 125.5 | 95 | 111.73 | 107.35 |
| Acetic acid (mM) | – | – | – | 17.12 | 17.12 | 7.25 | 7.25 | 7.25 | 7.25 | – | – | – | – | – | – | – |
| Sodium acetate (mM) | – | – | – | 29.75 | 29.75 | 64.65 | 64.65 | 64.65 | 64.65 | – | – | – | – | – | – | – |
| Maleic acid (mM) | – | – | – | – | – | – | – | – | – | 19.12 | 19.12 | 19.12 | 55.02 | 55.02 | 55.02 | 55.02 |
| Sodium hydroxide (mM) | – | – | – | – | – | – | – | – | – | 34.8 | 34.8 | 34.8 | 81.65 | 81.65 | 81.65 | 81.65 |
| Glyceryl monooleate (mM) | – | – | – | – | – | – | – | – | – | – | – | – | 5 | 5 | 6.65 | 5 |
| Sodium oleate (mM) | – | – | – | – | – | – | – | – | – | – | – | – | 0.8 | 0.8 | 1.06 | 0.8 |
| Milk: buffer | – | – | – | 1:1 | 1:1 | 1:1 | 1:1 | 1:1 | 1:1 | – | – | – | – | – | – | – |
| HCl/NaOH qs | pH 1.6 | pH 1.6 | pH 1.6 | pH 5 | pH 5 | pH 5.7 | pH 5.7 | pH 5.7 | pH 5.7 | pH 6.5 | pH 6.5 | pH 6.5 | pH 5.8 | pH 5.8 | pH 5.8 | pH 5.8 |
| pH | 1.6 | 1.6 | 1.6 | 5 | 5 | 5.7 | 5.7 | 5.7 | 5.7 | 6.5 | 6.5 | 6.5 | 5.8 | 5.8 | 5.8 | 5.8 |
| Osmolarity (mOsm/kg) | 120.7 | 120.7 | 120.7 | 400 | 400 | 340 | 340 | 240 | 240 | 180 | 180 | 180 | 390 | 300 | 330 | 330 |
| Buffering capacity (mEq/L/ΔpH) | – | – | – | 25 | 25 | 15 | 15 | 15 | 15 | 10 | 10 | 10 | 25 | 25 | 25 | 25 |

FaSSGF: Fasted-state simulated gastric fluid; Pn-FaSSGF: Pediatric fasted-state gastric media representative of neonates (birth to 27 days); Pi-FaSSGF: Pediatric fasted-state gastric media representative of infants (1–12 months); FeSSGF: Fed-state simulated gastric fluid; Pnc-FeSSGF: Pediatric fed-state gastric media representative of neonates (birth to 27 days) fed milk-based formula; Pns-FeSSGF: Pediatric fed-state gastric media representative of neonates (birth to 27 days) fed soy-based formula; FaSSIF.v2: Fasted-state simulated intestinal fluid; P-FaSSIF-50%: Pediatric fasted-state intestinal media formulated with bile salt concentrations 50% (i.e., 1.5 mM) of adult levels; P-FaSSIF-150%: Pediatric fasted-state intestinal media formulated with bile salt concentrations 150% (i.e., 4.5 mM) of adult levels; FeSSIF.v2: Fed-state simulated intestinal fluid; Pnb-FeSSIF: Pediatric fed-state intestinal media representative of neonates (birth to 27 days) fed breast milk; Pnc-FeSSIF: Pediatric fed-state intestinal media representative of neonates (birth to 27 days) fed milk-based formula; Pi-FeSSIF: Pediatric fed-state intestinal media representative of infants (1–12 months) fed milk-based formula.

**Supplementary Table S2:** The median weight, gastric volume, and normalized initial gastric volume in neonates, 6-month-old infants, infants, and children aging from 1 to 17 years.

| **Age (years)** | **Median weight (kg)^a^** | **Gastric volume (mL)^b^** | **Age-appropriate V_0_ (mL)^c^** | **Dosing per mg/kg** | **Dose in mg** |
| --- | --- | --- | --- | --- | --- |
| Newborn (neonate) | 3.95 | 2.21 | 14.9 | 15 | 59.25 |
| 0.5 (6-month-old infant) | 7.9 | 4.42 | 29.8 | 15 | 118.5 |
| 1 | 10.3 | 5.77 | 38.9 | 15 | 154.5 |
| 2 | 12.7 | 7.11 | 47.9 | 15 | 190.5 |
| 3 | 14.3 | 8.01 | 54 | 15 | 214.5 |
| 4 | 16 | 8.96 | 60.4 | 15 | 240 |
| 5 | 18.5 | 10.4 | 69.8 | 15 | 277.5 |
| 6 | 21 | 11.8 | 79.2 | 15 | 315 |
| 7 | 23 | 12.9 | 86.8 | 15 | 345 |
| 8 | 26 | 14.6 | 98.1 | 15 | 390 |
| 9 | 29 | 16.2 | 109 | 15 | 435 |
| 10 | 32 | 17.9 | 121 | 15 | 480 |
| 11 | 36 | 20.2 | 136 | 15 | 540 |
| 12 | 40.5 | 22.7 | 153 | 15 | 607.5 |
| 13 | 45.5 | 25.5 | 172 | 15 | 682.5 |
| 14 | 51 | 28.6 | 192 | 15 | 765 |
| 15 | 56 | 31.4 | 211 | 15 | 840 |
| 16 | 61 | 34.2 | 230 | 15 | 915 |
| 17 | 65 | 36.4 | 245 | 15 | 975 |

^a^The median weight for neonates, infants (aging 6 months), and infants and children of 1-17 years old were obtained from the growth charts of the Centers for Disease Control and Prevention [[3](#_ENREF_3)]

^b^The age-specific V_0_ values that correspond to that of adults (250 mL) based on the 0.56 mL/kg were calculated as described previously [[4](#_ENREF_4)]

^c^Age-specific V_0_ = ((median weight in kg) x 0.56 mL)/37.1 mL/kg)) x 250 mL

**Supplementary Table S3:** Molecular descriptors of lamotrigine

| **Descriptor** | **Value** |
| --- | --- |
| Experimentally determined LogP | 1.93 |
| Experimentally determined pKa | 5.7 |
| cLogP predicted by ALOGPS | 1.87 |
| cLogP predicted by ChemAxon | 1.93 |
| pKa (Strongest Acidic) predicted by ChemAxon | 14.98 |
| pKa (Strongest Basic) predicted by ChemAxon | 5.89 |
| Polar Surface Area predicted by ChemAxon | 90.71 Å^2^ |
| Bioavailability predicted by ChemAxon | 100% |





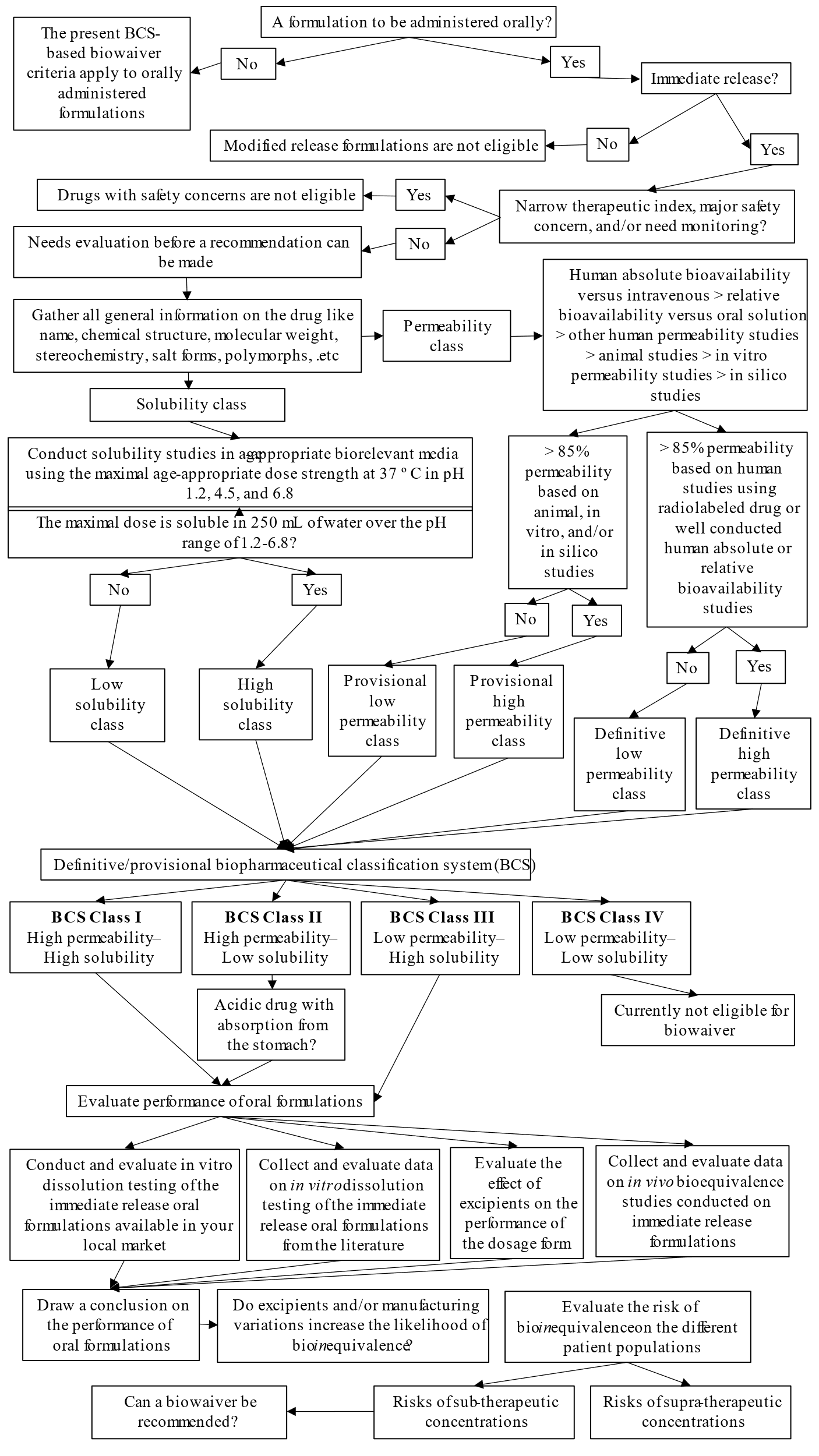


**Figure S2.** Flow diagram of a decision to recommend a biowaiver or not based on the guidelines of the BCS and biowaivers special interest group of the FIP


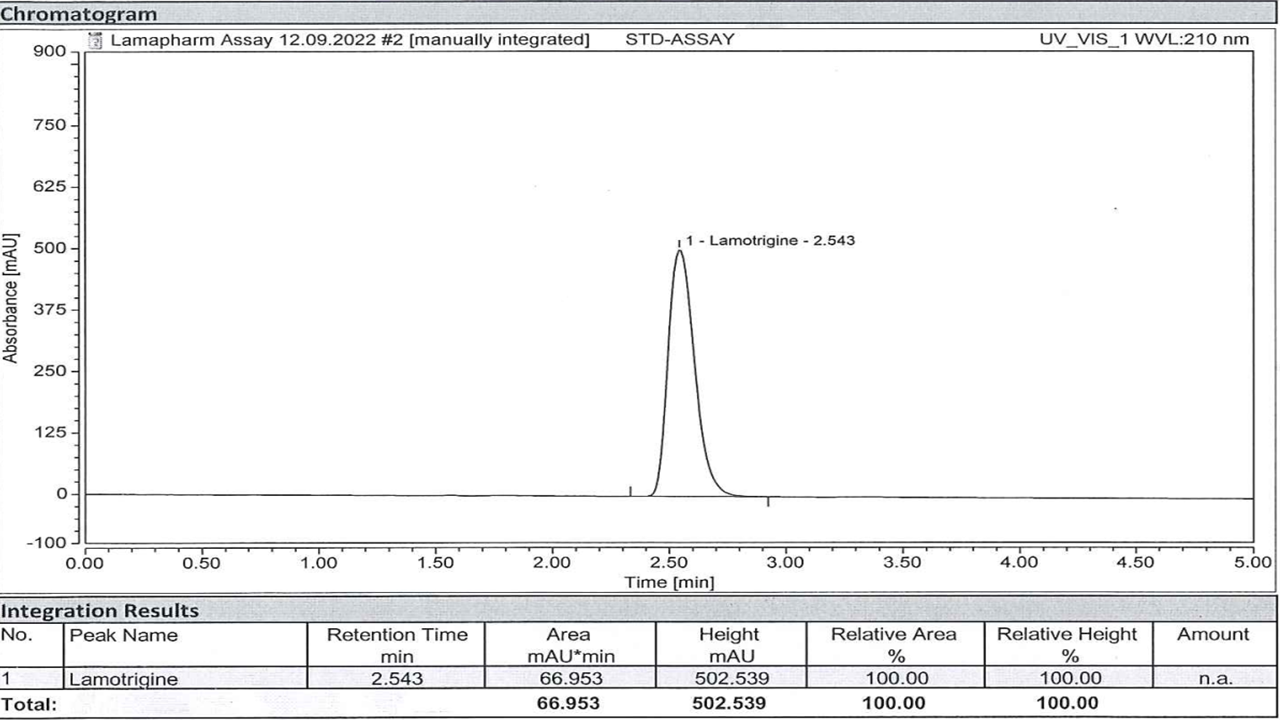
**Supplementary Figure S3:** Sample chromatogram

**References**

1. Maharaj AR, Edginton AN, Fotaki N: **Assessment of Age-Related Changes in Pediatric Gastrointestinal Solubility**. *Pharmaceutical Research* 2016, **33**(1):52-71.

2. Jantratid E, Janssen N, Reppas C, Dressman JB: **Dissolution media simulating conditions in the proximal human gastrointestinal tract: an update**. *Pharm Res* 2008, **25**(7):1663-1676.

3. (CDC) CfDCaP: **Stature-for-age and Weight-for-age percentiles. 2 to 20 years: Boys**. In*.*; 2000.

4. Shawahna R: **Pediatric Biopharmaceutical Classification System: Using Age-Appropriate Initial Gastric Volume**. *The AAPS journal* 2016, **18**(3):728-736.
